# COVID-19 infection during autoimmune disease: study of 2 cases in Republic of Guinea

**DOI:** 10.11604/pamj.supp.2020.35.2.24616

**Published:** 2020-07-01

**Authors:** Kaba Condé, Hugues Ghislain Atakla, Mahaman Salissou Garba, Idé Garba

**Affiliations:** 1 Neurology Department, Ignace Deen University Hospital Center, Conakry, Guinea; 2 Rheumatology Department, Ignace Deen University Hospital Center, Conakry, Guinea; 3 Rheumatology Department, Maradi National Hospital, Maradi, Niger

**Keywords:** COVID-19, rheumatoid arthritis, systemic scleroderma, Guinea

## Abstract

**Introduction:**

Coronavirus is a virus that can target the respiratory, musculoskeletal systems with a cascade of inflammatory processes. The objective of this work is to establish the link between autoimmune diseases and a COVID-19 infection in Guinea.

**Methods:**

Retrospective patient data were obtained from medical records. Informed consent was obtained under the direction of the national health security agency (ANSS).

**Results:**

We report the case of two patients aged 52 and 64 years respectively, known to have rheumatoid arthritis (RA) and systemic scleroderma (SDS) admitted with clinical signs suggesting underlying infection with COVID-19. They were tested with RT-PCR, which was positive within hours.

**Conclusion:**

In view of the rapid clinical worsening of patients with COVID-19 infection and autoimmune diseases, increased surveillance should be undertaken with abstinence of any factors that might weaken the immunity of these patients.

## Introduction

In December 2019, a new type of pneumonia due to the coronavirus epidemic was described in China. Very early we moved to a pandemic as described there by the World Health Organization on March 11, 2020 [1]. SARS-CoV-2 infection is characterized by dry cough, fever, dyspnea and fatigue, accompanied by other biological signs such as lymphopenia [2]. In more severe cases, the picture can be complicated by the development of interstitial pneumonia with alveolar involvement, which can clinically lead to acute respiratory distress syndrome (ARDS) and even death [3]. In this context of unprecedented health emergency declared by WHO, the infection with COVID-19 in patients already weakened by immuno-rheumatological diseases poses a real problem that deserves to be considered in a special chapter. However, the existence of autoimmune phenomena in patients with COVID-19 has not been reported. Viral effects and immune-mediated mechanisms are the two pathogens of coronavirus infection associated with severe acute respiratory syndrome (SARS-Cov), and autoimmune responses have been found in SARS-Cov infection [4]. Wang Y et al. have suggested that CoV-SARS antigen cross-reacts with autoantibodies in autoimmune diseases [5]. Drugs commonly used for the treatment of rheumatoid arthritis (RA), are now being used even for the management of more complex cases of COVID-19. Chloroquine and hydroxychloroquine have now been permanently included, alongside antiviral drugs, in treatment protocols for pneumonia caused by COVID-19 [6]. In the present study, we present the clinical, autoimmune and biological characteristics of COVID-19 caused by CoV-2-SARS in patients known and followed for systemic disease. The objective of the work is to establish the link between autoimmune diseases and COVID-19 infection in Guinea.

## Methods

Retrospective patient data were obtained from medical records. Informed consent from patients and/or parents was obtained in agreement with the National Health Safety Agency (ANSS). We report in this work the case of 2 patients known to have autoimmune disease and infection with the new coronavirus.

## Results

**Figure 1:**
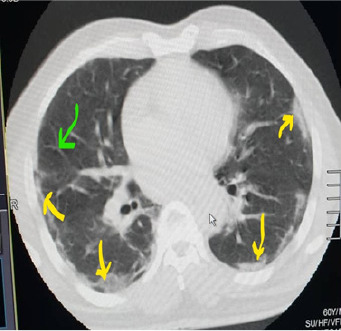
Frosted glass opacifications posterior basal peripheral and bilateral (yellow arrow); thickening of the interlobral septa (green arrow)

**Case n°1:** we report a 52-year-old patient known to have rheumatoid arthritis since 2012, meeting the American college of rheumatology (ACR)/European league against rheumatism (EULAR) 2010 criteria and treated with methotrexate 20mg/week and prednisolone 10mg daily. In April 2020, he was admitted to the emergency department of Ignace Deen National Hospital for a flare-up of his rheumatoid arthritis with a disease activity score-8 to 5.2 (DAS-28 at 5.2) associated with an influenza-like illness, a fever of 38.2°C with difficulty breathing and a cough. The patient is unaware of whether or not he has been in contact with a coronavirus carrier. Altered general condition and quality of life were noted with a Health assessment questionnaire (HAQ) of 15/60, blood pressure at 130/80mmhg, heart rate (HR) at 111 beats per minute; oxygen saturation at 83%. Physical examination showed inflammatory synovitis in the wrists; at metacarpophalangeal 2 and 3; knees bilaterally. Cardiopulmonary examination showed tachycardia associated with crackling rales at both lung bases. The rest of the examination was unremarkable. The biological examination showed a normal blood count, a non-specific inflammatory syndrome with a sedimentation rate (SV) of 52mm/h and a C-reactive protein (CRP) of 35mg/l. Renal and liver function was normal. Cytobacteriological examination of the urine and blood culture were negative on 2 occasions. Chest X-ray showed a mundane interstitial lung disease. Thoracic CT showed lesions suggestive of COVID-19, i.e., frosted glass opacifications, posterior basal peripheral and bilateral with thickening of the interlobar septa (Figure 1). The patient was placed in isolation and placed on the standard treatment adopted by Guinea (Chloroquine; Azythromycin) and corticosteroid therapy and methotrexate was discontinued. The COVID-19 PCR confirmatory test that was performed on admission was later (24 hours) positive. The D-dimer level was 2120μg/L. Thus the diagnosis of COVID-19 infection associated with rheumatoid arthritis was retained. The patient was discharged 33 days later following regression of symptoms and the RT-PCR test was negative on two occasions.

**Figure 2:**
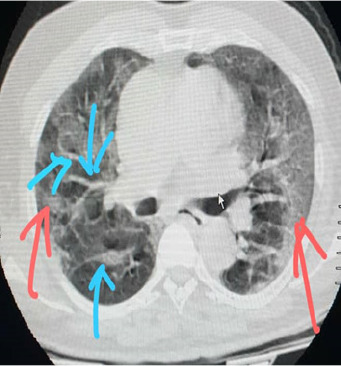
Predominantly peripheral and posterior bilateral multifocal opacities (red arrow); thickening of interlobral sept (blue arrow)

**Case n°2:** This was a 64-year-old patient with a history of diabetes and hypertension known to have limited systemic scleroderma according to ACR/EULAR 2013 criteria since 2015. She was being treated with calcium channel blockers and oral antidiabetic agents. In May 2020, she was admitted to the National Center for Infectious Diseases for cough, dyspnea with 66% oxygen desaturation; fever at 40°C associated with hypersudation. The anamnesis found a notion of contagion with four members of her family who tested positive for COVID-19. Physical examination revealed an alteration in general condition; tachycardia; tachypnea with crackling rales in both lung fields. CT showed bilateral multifocal opacities predominantly peripheral and posterior with thickening of the interlobar septa (Figure 2). Treatment with chloroquine and Azythromycin was instituted. Bioassay showed a blood count within normal limits; a biologic inflammatory syndrome with a SV at 66 mm/h and one at 36 mg/l. Renal function was normal. Eighteen (18) hours after admission, the patient was confirmed positive for COVID-19. The diagnosis of COVID-19 in the field of scleroderma was retained. The course was marked by worsening respiratory symptoms and the patient died 71 hours after admission.

## Discussion

Few studies have examined a potential link between viral respiratory infections and the development of autoimmune diseases such as RA [7]. To date, very little data is available on chronic inflammatory rheumatic diseases and COVID-19 [7]. Rheumatoid arthritis (RA) is associated with an increased risk of infection compared to the general population without RA [8]. As for scleroderma, pre-existing lung involvement in this disease may aggravate a COVID-19 infection. However, to our knowledge, this is the first case of COVID-19 in scleroderma reported in the literature. The authors collected information on 2 patients (1 male and 1 female) aged 52 and 64 years respectively. There is currently very little data available regarding the gender distribution in patients with coronavirus infection in systemic disease field. However, Listing J and Col. Report that there is an increased risk of infection related to advanced age and female sex [9]. Our patients were all clinically symptomatic at COVID-19 and the notion of contagion was found in a patient with several close family members confirmed positive for coronavirus. This situation therefore represents a predilection for opportunistic diseases for patients who suffer from systemic disease and are undergoing immunosuppressive treatment [10]. The existence of RA and SDS in the two patients associated with COVID-19 infection appears to be a vicious circle since RA and SDS are all systemic inflammatory diseases in the same way as COVID-19 viral aggression. This probably explains the presence of the biological inflammatory syndrome found in both patients. The chest CT scan routinely performed for the respiratory distress syndrome isolated the two patients while awaiting confirmation of COVID-19 infection by RT-PCR.

The two patients presented in this work were undergoing corticosteroid therapy at one prior to admission. It should be noted that anti-rheumatic drugs are responsible for an increased risk of infection related not only to advanced age and female sex but also to treatment with prednisolone at a dose greater than 7.5 mg/day and a high number of hospital stays [9] The occurrence of COVID-19 infection in these patients could be explained by the decrease in cellular immunity associated with corticosteroid use. On the basis of the action of chloroquine on COVID-19 and its efficacy demonstrated by several studies [11-13], all patients received treatment with Chloroquine 1000mg/day and Azythromycin 500mg (standard treatment adopted in our context in Guinea). Considering the experience of Dieudonné Ouédraogo et al. on the use of Chloroquine/hydroxychloroquine in the treatment of autoimmune diseases (systemic lupus erythematosus; rheumatoid arthritis; scleroderma and others), this standard protocol that we adopted in these patients could have a double beneficial effect (on COVID-19 infection; RA and SDS). The outcome was the recovery of patient 1 and the death of patient 2. Until further studies clarify the absence of higher risk in patients with chronic inflammatory rheumatic diseases, increased surveillance of these patients is necessary and the application of barrier gestures is useful for all.

## Conclusion

Aggravation of autoimmune phenomena exists in COVID-19 subjects, and this finding provides a rationale for a strategy to prevent immune dysfunction and optimize immunosuppressive therapy in the future. Discontinuation of corticosteroid therapy in these patients could promote recovery of their immunity and limit the occurrence of opportunistic infections. Further studies are likely to be needed to characterize this patient group.

### What is known about this topic

- Subjects infected with COVID-19 show respiratory and systemic symptoms.

### What this study adds

- Aggravation of autoimmune phenomena in subjects infected with COVID-19.

## Competing interests

The authors declare no competing interests.
